# Evaluation of different decontamination procedures on bond strength to sound and caries affected dentin using “no-wait” universal adhesive

**DOI:** 10.1186/s12903-023-03314-2

**Published:** 2023-09-05

**Authors:** Lamiaa M Moharam, Haidy N Salem, Sherif Khadr, Ahmed Abdou

**Affiliations:** 1 Restorative and Dental Materials Department, Oral and Dental Research Institute, National Research Centre, Giza, Dokki, 12622 Egypt; 2 School of Dentistry, Newgiza University, Giza, Egypt; 3 Conservative Dentistry Department, Faculty of Oral and Dental Medicine, Future University, Cairo, Egypt; 4 Prosthetic Dentistry Department, Faculty of Dentistry, King Salman International University, El Tur, South Sinai, Egypt

**Keywords:** Contamination, Decontamination procedures, Bonding stages, Bond strength, Sound dentin, Caries affected dentin, No-wait, Universal adhesive

## Abstract

**Background:**

Current study aimed to evaluate the effect of different decontamination procedures on micro-shear bond strength (μSBS) of sound (SoD) and caries-affected dentin (CAD) of two universal adhesives after blood-saliva contamination.

**Methods:**

One hundred and eighty bovine anterior teeth were prepared and allocated into the respective groups according to tested dentin substrates [SoD, CAD], universal adhesives [Clearfil Bond Universal Quick (UBQ), All-Bond-Universal (ABU)], adhesive contamination stage [none, contamination before and after adhesives light-curing], and according to decontamination procedures [no decontamination, water rinsing, adhesive rebond, Ethylenediaminetetraacetic acid (ETDA) and chlorhexidine (CHX) application]. Universal adhesives were applied according to manufacturer instructions in self-etch (SE) bonding mode. Four composite microrods were built for each tooth. Specimens were kept in distilled water for 24 hours at 37°C before testing μSBS. Four-way ANOVA and Tukey HSD tests were used for data analysis.

**Results:**

A statistically significant difference between contamination stages of both universal adhesives at different decontamination procedures for SoD and CAD. Highest μSBS was recorded for UBQ control group at SoD, while the least was recorded for light-cured ABU upon water rinsing decontamination procedure of CAD.

**Conclusions:**

Proper cavity isolation is mandatory to avoid possible contamination which can dramatically affect μSBS. CHX is a potent cavity decontaminant that can restore different dentin substrates bond strength. EDTA presents a promising substitute. UBQ adhesive showed better bonding performance than ABU to both dentin substrates. Application of regular cavity decontamination approaches is highly advised in daily practice to avoid possible detrimental effect of accidental cavity contamination.

## Background

The ‘universal’ or ‘multimode’ adhesives were introduced as the recent generations of the contemporary one-step SE adhesives. The manufacturers claimed that such adhesives are effectively able to bond to enamel and dentin in etch-and-rinse (ER) and SE modes. This new generation of multi-mode adhesives has by now demonstrated a positive instant clinical performance compared to that of the gold standard ER and SE contemporary adhesives [1, 2]. These adhesives have a durable bonding to enamel and dentin as well as various restorative materials with appropriate handling of their surfaces [2–4]. Such exceptional features could be owed to the integration of 10-methacryloyloxydecyl dihydrogen phosphate (10-MDP) adhesive monomer in their composition [5].

Furthermore, a novel category of universal adhesives has been recently developed with the ‘quick bonding’ or ‘no-wait’ concept. These adhesives could be applied in SE bonding mode without waiting. Accordingly, these adhesives offer less technique sensitivity and simplify the bonding procedures for dental practitioners and can be applied with no time to wait for the application of the adhesive [6]. While an application time of shorter duration is clinically satisfactory, it could yield adverse effects on the bonding performance of such adhesives [7]. Yet, Huang et al. [8] assessed the dentin bonding strength of no-wait and SE adhesives applied in no-waiting or 10 seconds of SE bonding approach, and they reported an adequate dentin bond strength with the “no-waiting” universal adhesives in the SE mode. However, 10 seconds waiting time showed a higher dentin bond strength with SE bonding mode.

A long-term successful restoration can be achieved via proper clinicians’ orientation of the adhesive mechanism, besides the proper application of the adhesive systems [3, 9]. Nevertheless, the major objectives of contemporary dental adhesives are to diminish dentin bonding difficulties and to simplify the bonding procedures. Although SoD is usually involved in in-vitro studies, bonding to CAD is more frequent in the daily clinical practice [10]. Clinically, dental caries management may include the elimination of the necrotic and infected carious tissues with preservation of the remaining affected carious structures followed by the appropriate adhesive procedures [11]. Dental caries can lead to serious structural changes to dentin including increased porosity of inter-tubular dentin, mineral loss, hydroxyapatite crystals dissolution, as well as enzymatic and bacterial degradation of the exposed collagen fibrils, which might undesirably influence the bonding to CAD leading to inferior dentin hybridization compared to SoD [12] and thus affecting its durability [10].

Clinically, dentin bond strength is jeopardized by contamination due to seepage of sulcular fluid, water, blood, and saliva into the prepared cavity upon inappropriate field isolation [13]. When the prepared cavity is contaminated with blood, a layer composed of blood protein, platelets and fibrinogen will be formed on the dentin surface, hindering the adhesive infiltration within the dentinal tubules [14]. Moreover, the blood protein will react with the exposed dentin collagen fibrils, interfering with the chemical bonding to dentin. As dentin contamination before using universal adhesives could negatively affect the dentin bond strength, particularly when such adhesives are employed in SE bonding mode, multiple reliable decontamination approaches could be employed depending on the adhesive system used such as water rinsing, re-bonding using a layer of the adhesive, re-etching with phosphoric acid, or using CHX, or ETDA [15] to counteract the adverse effects of saliva and blood contamination [16]. The ultimate dentin decontamination should have an effective antimicrobial activity without interference with the adhesion process. Additionally, applying different cavity decontaminants prior to SE adhesives could be crucial owing to the lack of the post-acid etching and rising step and the inability to eliminate smear layer completely. Therefore, few products were proposed as cavity decontaminants with acceptable outcomes. CHX is the most employed cavity decontaminant, which has a potent antimicrobial action on dental caries, and it is capable of impeding the acquired enamel pellicle formation, thus preventing dental plaque development [17]. Moreover, maintained bond strength values were reported upon dental cavity decontamination using CHX at any assessed concentration, which could be related to its capacity to restrain matrix metalloproteinases (MMPs) responsible for adhesive interfaces degradation, thus showing enhanced bond strength outcomes [18]. Still, the influence of cavity decontaminants on dentin bonding is yet to be ambiguous for the majority of these agents [11].

Since the studies concerned with efficient decontamination approaches for various categories of universal adhesives are infrequent, there is an urge for further studies to assess the efficacy of different decontamination methods with different universal adhesives. Thus, the present study aimed to evaluate the effect of different contamination and decontamination procedures at different bonding stages on the bond strength of sound and caries affected dentin using ‘No-Wait’ universal adhesive. The null hypotheses were: (a) The contamination and different decontamination procedures would have no effect on dentin bonding of SoD and CAD (b) The tested universal adhesives would have no effect on the bonding performance of SoD and CAD. (c) The contamination stages of the tested universal adhesives would have no effect on dentin adhesion of SoD and CAD at the different decontamination procedures.

## Methods

### Ethical approval

This study was approved by the Medical Research Ethical Committee (MREC) of the National Research Centre (NRC); Giza, Egypt; under the reference number: 0117082022.

### Selected materials

Two universal adhesives; [All-Bond Universal (ABU: BISCO, Inc., Schaumburg, IL, USA) and Clearfil Universal Bond Quick (UBQ: Kuraray Noritake Dental Inc., Okayama, Japan)], two cavity disinfectants; [2% Chlorhexidine gluconate (CHX: Consepsis, Ultradent Products Inc., South Jordan, UT, USA) and 17% Ethylenediaminetetraacetic acid (EDTA: MD-Cleanser, METABIOMED Co., LTD, Cheongju City, Chungbuk, Korea) solutions], and a nanohybrid flowable resin composite [Filtek™ Z350 XT (3 M Oral Care, St. Paul, MN, USA) Flowable Restorative] were used in this study. The materials brand name, description, composition, and their manufacturers are listed in Table 1.

**Table 1:** The materials used in the study and their composition, description, and manufacturer

Material		Description	Composition	Manufacturer
Consepsis™		Cavity disinfectant and smear layer removing solution	2% Chlorhexidine Gluconate, Ethyl Alcohol, Polyethylene Glycol, Dimethicone, Oils, Peppermint flavor	Ultradent Products Inc., South Jordan, UT, USA
MD-Cleanser		Root canal cleaning and smear layer removing solution	17% EDTA, water	METABIOMED Co., LTD, Cheongju City, Chungbuk, Korea
All-Bond Universal (ABU)		Universal adhesive	Bis-GMA, ethanol, HEMA, 10-MDP	BISCO, Inc., Schaumburg, IL, USA
Clearfil Universal Bond Quick (UBQ)		‘No-Wait’ Universal adhesive	Bis-GMA, ethanol, HEMA, 10-MDP, Hydrophilic amide monomers, Colloidal silica, Silane coupling agent, Sodium fluoride, dl-Camphorquinone, Water	Kuraray Noritake Dental Inc., Okayama, Japan
Filtek™ Z350 XT Flowable Restorative		Nanohybrid flowable resin composite restorative material	Bis-GMA, TEGDMA, Procrylat resins, ytterbium trifluoride filler (0.1-5.0 microns), Silica (20-nm non-agglomerated/aggregated), silica (75-nm non-agglomerated/aggregated and agglomerated), clusters fillers of zirconia/silica aggregated particles (20 nm silica particles combined with 4–11 nm zirconia)	3 M Oral Care, St. Paul, MN, USA

EDTA: Ethylenediaminetetraacetic acid. HEMA: Hydroxyethyl methacrylate. MDP: Methacryloyloxydecyl dihydrogen phosphate. Bis-GMA: Bisphenol A diglycidyl methacrylate. UDMA: Urethane dimethacrylate, TEGDMA: Triethylene glycol dimethacrylate

### Teeth selection

One hundred and eighty upper anterior bovine teeth were collected for the current study. The teeth were cleansed from any residual debris or soft tissues under running tap-water with a sharp hand scaler. The selected teeth were examined under x25 magnifying lens to exclude any cracked, fractured, or defective teeth. Then the teeth were reserved at 4 °C in 0.1% thymol solution up to three months maximum period after extraction with changing the solution once per week till use [19].

### Tooth specimens’ preparation

Teeth roots were cut 2-mm below the cementoenamel junction using a double-side diamond disc mounted to a low-speed handpiece. The pulp chamber contents were removed with barbed broaches [2]. The labial enamel was ground under wet condition using 240-grit silicon carbide (SiC) abrasive paper to expose the underlying midcoronal dentin. Using wet SiC 600-grit paper, the exposed dentin surfaces were finished for one minute in a circular motion to produce a standardized smear layer [2, 20]. The specimens were inspected using stereomicroscope (Olympus® BX 60, Olympus Optical Co. LTD, Tokyo, Japan) to detect enamel residues or further defects. The specimens were fixed in chemical cure acrylic resin blocks [21] and the specimens were instantly kept in distilled water after complete polymerization of the acrylic resin [22].

### Experimental design of the study

Sample size was calculated based on Elkassas and Arafa 2016 [23]. The difference between uncontaminated (Control) and decontaminated + adhesive rebond was 12.9 and the standard deviation was 1.6 and 3.01, respectively. The minimum sample size in each group will be three teeth with effect size (d = 5.36), the α = 0.05 and will result in 95% power. Sample size increased to five teeth in each group for statistical analysis reliability.

**Fig. 1:**
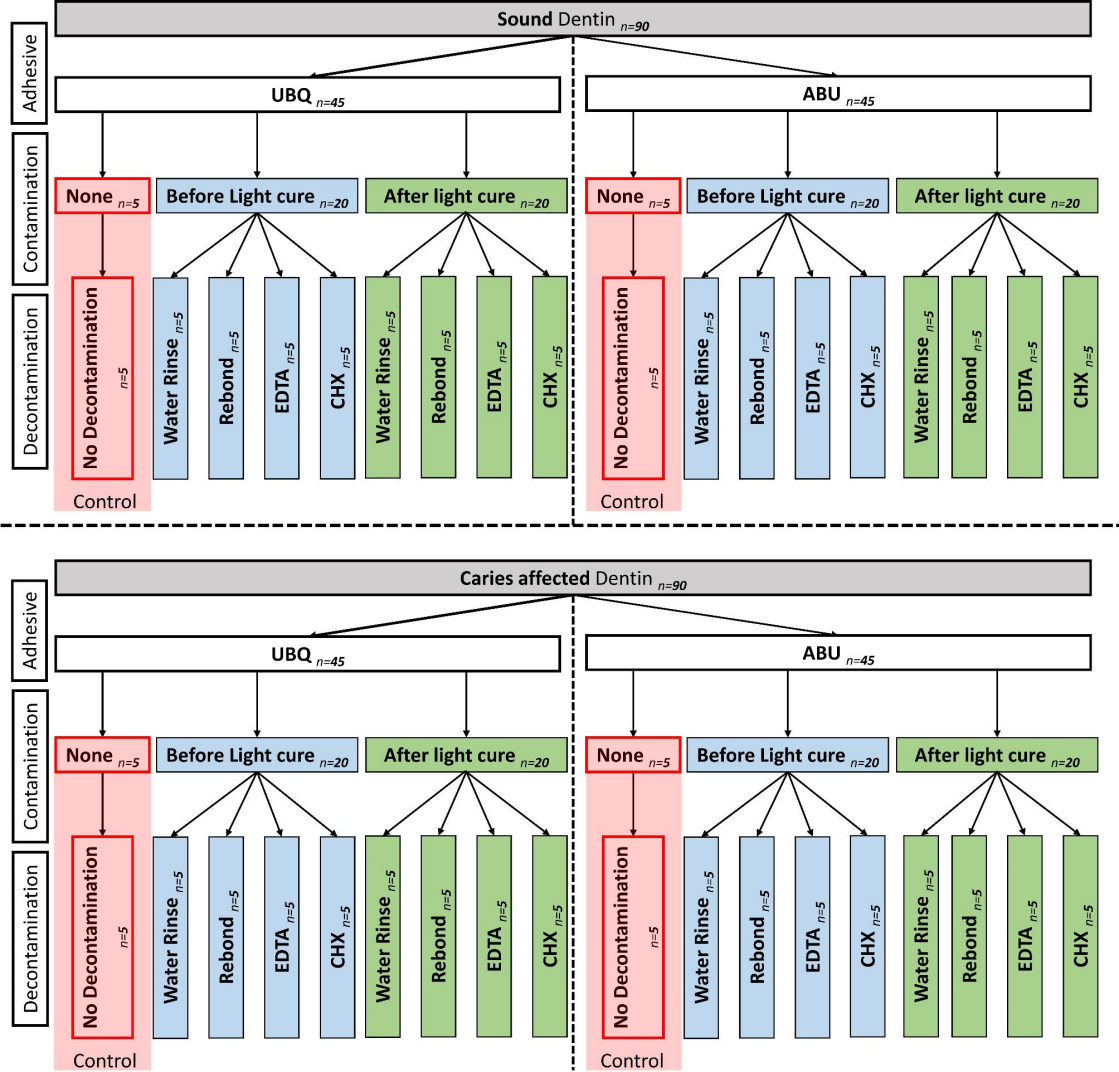
Flowchart showing the experimental design and grouping of the present study

Specimens grouping, frequency and study design are presented in Fig. 1. A total of 180 teeth were divided into two groups according to type of dentin substrate into: sound dentin (SoD, n = 90) and caries-affected dentin (CAD, n = 90). Each group was divided into two subgroups according to the universal adhesives used into: UBQ (n = 45) and ABU (n = 45). Then, each subgroup was divided according to the contamination at various stages of adhesive application into:

1. None (n = 5): the universal adhesives were light cured according to the manufacture instruction.
2. Before light-curing of the adhesive systems (n = 20): contamination with blood and saliva mixture was applied for 20 seconds before light-curing of the adhesive system.
3. After light-curing of the adhesive systems (n = 20): contamination with blood and saliva mixture was applied after light-curing of the adhesive systems.

Furthermore, each sub-subgroup was divided according to the decontamination procedures into:

1. No decontamination (n = 5): the “None” sub-subgroup was light cured according to manufacturer instruction with no decontamination procedure.
2. Water rinsing (n = 5): rinsing with water stream for 30 seconds.
3. Adhesive rebond (n = 5): rinsing with water stream for 30 seconds, followed by reapplication of the tested universal adhesives.
4. EDTA application (n = 5): rinsing with water stream for 30 seconds, followed EDTA application for 15 seconds followed by water rinsing for 30 seconds, followed by reapplication of the tested universal adhesives.
5. CHX application (n = 5): rinsing with water stream for 30 seconds followed CHX application for 15 seconds followed by water rinsing for 30 seconds, followed by reapplication of the tested universal adhesives.

The None sub-group and No decontamination sub-subgroup is considered as control (n = 5) as explained in Fig. 1.

### Caries-Affected dentin (CAD) production

Cariogenic challenge was employed to produce the artificial caries-affected lesions of dentin. Half of specimens (n = 90) were exposed to pH cycling protocol using prepared demineralizing and remineralizing solutions according to the procedure proposed by Nicoloso et al. 2017 [24].

### Application of universal adhesives

Both tested universal adhesives (ABU and UBQ) were applied to SoD and CAD using SE bonding technique according to manufacturers’ instructions. Two coats of ABU were applied with scrubbing action to dentin with micro brushes for ten to 15 seconds for each coat without light curing between the coats [2]. The excess solvent was evaporated by thorough air-drying using air syringe for ten seconds, until there was no visible movement of the adhesive [2]. Furthermore, UBQ was applied with a rubbing motion to the dentin substrates using micro brushes without any waiting time followed by mild air blow for five seconds.

### Contamination and decontamination procedures

A mixture of equivalent portions of blood and saliva was prepared from fresh unstimulated saliva collected two hours after breakfast, and fresh blood from needle-punctured fingertip. The blood and saliva were collected from a single investigator throughout the study [16]. Both universal adhesives were applied to SoD and CAD according to their manufacturers’ instructions without the application of blood-saliva mixture contamination presenting the control groups (Fig. 1).

Before light-curing of the applied universal adhesives of the experimental groups; both universal adhesives were applied to SoD and CAD according to their manufacturers’ instructions, then a uniform blood-saliva mixture contamination of the specimens was done for 20 seconds prior to light curing of the tested universal adhesives for ten seconds using LED light curing unit (Elipar S10, 3 M ESPE, USA). On the other hand, after light-curing of the applied universal adhesives of the experimental groups; the universal adhesives were applied to the SoD and CAD then light cured with the LED curing unit for ten seconds followed by the application of the uniform blood-saliva mixture contamination for 20 seconds. The LED light curing unit had an intensity ≥ 1000 mW/cm^2^, which was inspected sporadically with a handheld radiometer (Demetron 100, Kerr Corporation, CA, USA). Then the five decontamination procedures were applied [no decontamination, water rinsing, adhesive rebond, ETDA and CHX application].

### Resin composite microrods build-up

Tygon tubes of 0.8 mm internal diameter and 2-mm height were cut using a sharp scalpel then Filtek Z350 nanohybrid flowable resin composite was used for microrods build-up [2]. Each specimen received four microrods (n = 20/group). As per the manufacturer’s commands, each microrod was light cured for 10 seconds using the LED light curing unit. Specimens were kept in distilled water in tightly sealed plastic containers at 37 °C for 24 hours until the μSBS was assessed [21, 22].

### Micro-shear bond strength testing (μSBS) and failure mode assessment

Specimens were attached to the lower jig of a universal testing machine (Instron®, Model 3345, Instron Industrial Products, Canton, MA, USA) then a loop of stainless-steel wire (0.8 mm) was attached to the upper jig of the universal testing machine. The wire was placed around each composite microrod that was loaded with shear force until fracture with 5-kN load cell. The test was run at 1-mm/min cross head speed. The average of four composite microrods values per single tooth specimen denoted the value of each specimen. The data was recorded using computer software (Instron® Bluehill Lite Software, Instron Testing Software, Norwood, MA, USA). The μSBS was calculated in megapascals (MPa) by dividing the maximum load in Newtons (N) by the cross-sectional area of the bonded surface in mm^2^. Debonded specimens were investigated with the stereomicroscope at x35 magnification. Failure modes were categorized as cohesive when the failure was detected within the resin composite or the dentin substrates, adhesive if the failure was identified at the composite/tooth interface, and mixed when cohesive and adhesive fractures were identified concurrently. The pretest failure was recorded for the tested specimens, and it was considered as adhesive failure. The average of four composite microrods were calculated for each tooth and was considered as the statistical unit. The pretest failure was recorded as zero. However, any specimen that recorded more than two pretest failures was discarded and replaced with another specimen.

### Statistical analysis

The data was explored for normality and homoscedasticity assumptions using Kolmogorov–Smirnov test and Levene’s test, respectively. Data Showed a normal distribution and Levene’s test showed that equal variance, F (35, 180) = 1.167, *p* = 0.256. Four-way ANOVA was used to show the effect of the dentin substrate [SoD vs. CAD], the type of universal adhesive [ABU vs. UBQ], contamination stages of the universal adhesives’ application procedure [control group (none), before light curing, and after light curing of the adhesives], and the decontamination procedures [no decontamination, water rinsing, adhesive rebond, EDTA, and CHX application] on the μSBS. Tukey’s HSD test was used for multiple comparisons. The significance level was set at *p* < 0.05. Statistical analysis was performed with IBM SPSS Statistics Version 20 for Windows (IBM Documentation products, Armonk, NY, USA).

## Results

**Fig. 2:**
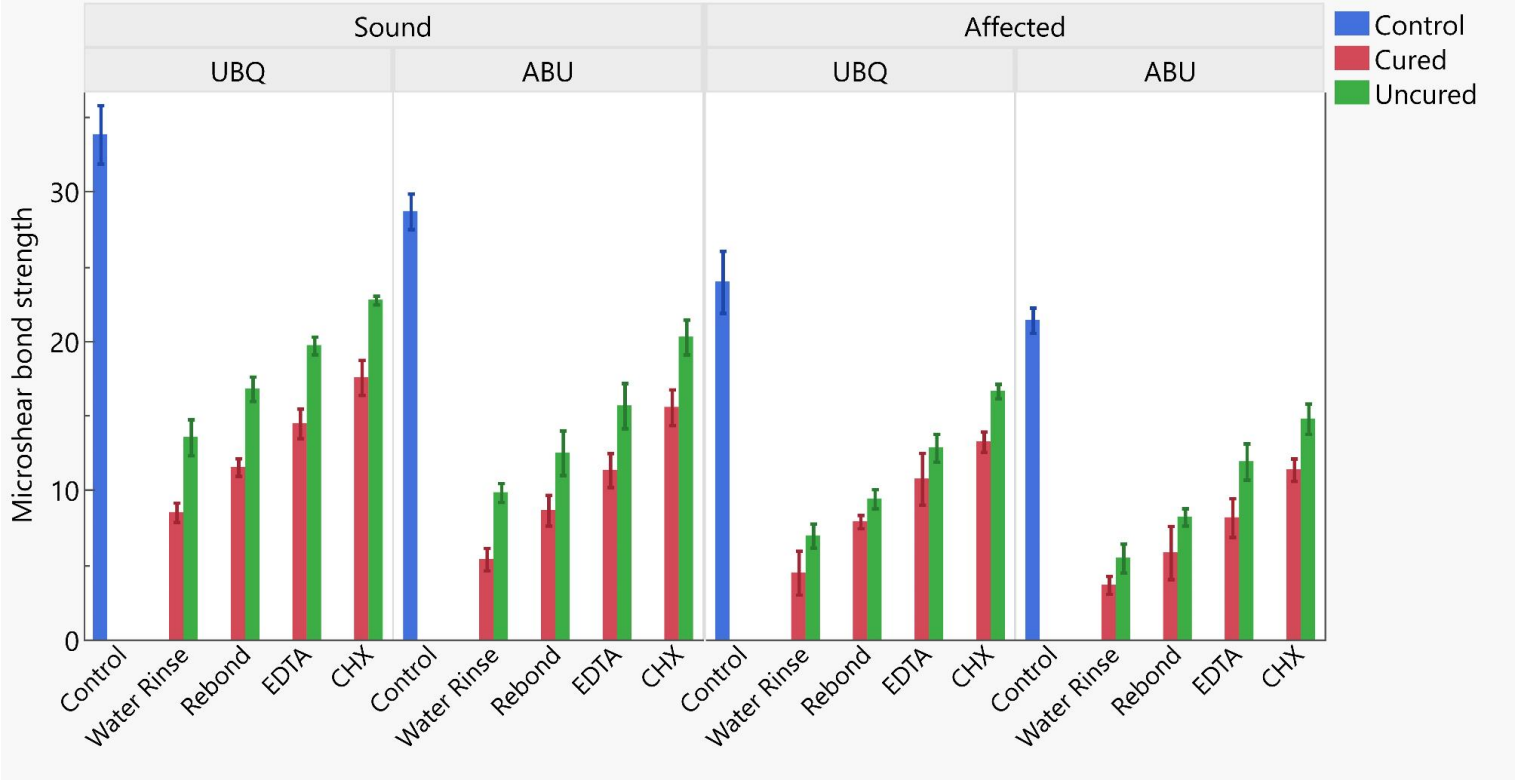
Bar chart for the μSBS of the different tested groups

**Fig. 3:**
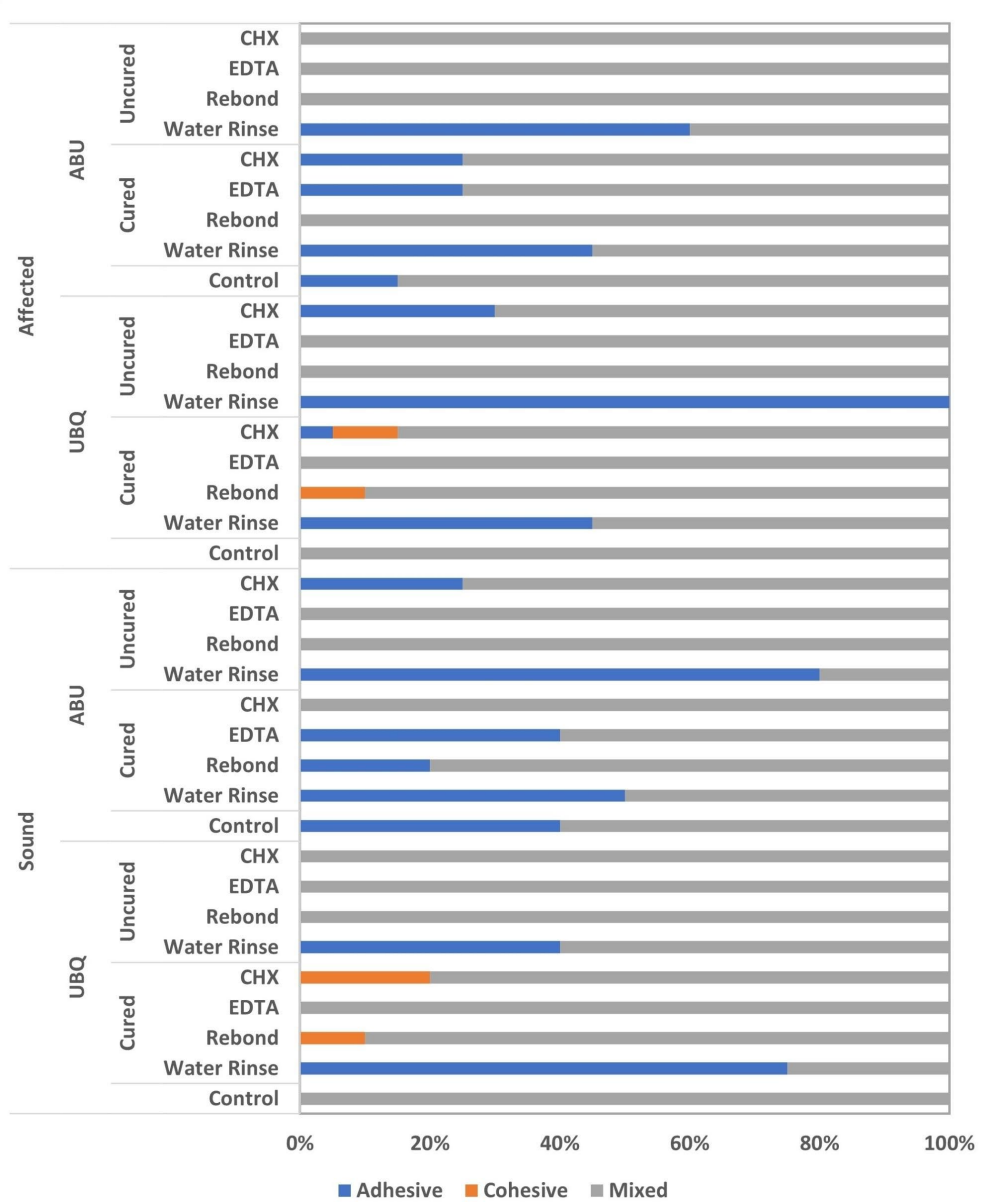
Stacked bar chart presenting the failure modes of the different tested groups

Four-way ANOVA analysis is presented in Table 2. The tested variables showed a statistically significant difference between the decontamination procedures, universal adhesive type, contamination stages of the universal adhesives’ application procedure, and the dentin substrates at *p* < 0.001. The interaction between all four variables had a statistically insignificant effect on the μSBS at *p* = 0.369. Table 3; Fig. 2 showed the effect of the different decontamination procedures on the μSBS of the tested groups. The results revealed a statistically significant difference between the control groups of both adhesives to SoD, while no statistically significant difference was recorded for both adhesives to CAD. For both dentin substrates, control groups showed the highest significant μSBS compared to the contaminated groups (*p* < 0.05) and no decontamination protocol applied was able to restore the μSBS. There was a statistically significant difference between the contaminated groups before and after light curing of the two universal adhesives at the different decontamination procedures for both dentin substrates (*p* < 0.05). For both universal adhesives [contaminated before and after light curing] and both dentin substrates, CHX application showed the highest significant μSBS Followed by EDTA application followed by the adhesive rebond application groups, and the least μSBS was reported for the water rinse group. For SoD, UBQ showed the highest significant μSBS compared to ABU for all tested groups (*p* < 0.05). For CAD, an insignificant difference resulted between UBQ and ABU for water rinse groups of both contaminated light-cured adhesives. All adhesive groups contaminated before light curing showed an insignificant difference between UBQ and ABU with all decontamination groups except CHX group, which showed a significantly higher μSBS for UBQ compared to ABU. For the failure mode analysis results, all the groups showed a predominate mixed failure. However, the adhesive failure was observed in the water rinse group within other variables (Fig. 3).

**Table 2:** Four-way ANOVA analysis for the different investigated variables of the current study

Source	Type III Sum of Squares	df	Mean Square	F	Sig.
Dentin Substrates	1409.817	1	1409.817	1143.005	< 0.001
Universal Adhesives	355.461	1	355.461	288.189	< 0.001
Contamination stages	647.222	1	647.222	524.734	< 0.001
Decontamination procedures	2281.563	3	760.521	616.590	< 0.001
Dentin Substrates × Universal Adhesives	39.486	1	39.486	32.013	< 0.001
Dentin Substrates × Contamination stages	55.161	1	55.161	44.721	< 0.001
Dentin Substrates × Decontamination procedures	4.533	3	1.511	1.225	0.302
Universal Adhesives × contamination stage	0.411	1	0.411	0.333	0.565
Universal Adhesives × Decontamination procedures	3.036	3	1.012	0.820	0.484
Contamination stages × Decontamination procedures	6.042	3	2.014	1.633	0.183
Dentin Substrates × Universal Adhesives × Contamination stages	5.098	1	5.098	4.133	0.044
Dentin Substrates × Universal Adhesives × Decontamination procedures	6.278	3	2.093	1.697	0.169
Dentin Substrates × Contamination stages × Decontamination procedures	2.541	3	0.847	0.687	0.561
Universal Adhesives × Contamination stages × Decontamination procedures	1.569	3	0.523	0.424	0.736
Dentin Substrates × Universal Adhesives × Contamination stages × Decontamination procedures	3.910	3	1.303	1.057	0.369

**Table 3:** Mean and standard deviation (SD) values of μSBS of the different tested groups

		**Sound Dentin (SoD)**		Caries-Affected Dentin (CAD)		*p*-value
		UBQ	**ABU**	UBQ	**ABU**	
**Control**		33.83^aA^ ± 1.96	28.68^aB^ ± 1.19	23.96^aC^ ± 2.32	21.39^aC^ ± 0.84	< 0.001
**Contaminated adhesives after light curing**	**Water rinse**	8.53^gA^ ± 0.65	5.39^gB^ ± 0.75	4.49^hBC^ ± 1.47	3.67^fC^ ± 0.6	< 0.001
	**Rebond**	11.56^fA^ ± 0.59	8.67^fB^ ± 1.02	7.91^fgB^ ± 0.45	5.84^eC^ ± 1.79	< 0.001
	**EDTA**	14.48^eA^ ± 0.99	11.36^deB^ ± 1.14	10.77^deB^ ± 1.73	8.18^dC^ ± 1.3	< 0.001
	**CHX**	17.56^dA^ ± 1.17	15.57^cB^ ± 1.2	13.26^cC^ ± 0.68	11.39^cD^ ± 0.75	< 0.001
**Contaminated adhesives before light curing**	**Water rinse**	13.56^eA^ ± 1.2	9.85^efB^ ± 0.64	6.97^gC^ ± 0.81	5.47^efC^ ± 0.97	< 0.001
	**Rebond**	16.8^dA^ ±0.81	12.51^dB^ ± 1.5	9.44^efC^ ± 0.65	8.22^dC^ ± 0.58	< 0.001
	**EDTA**	19.7^cA^ ± 0.59	15.67^cB^ ± 1.51	12.85^cdC^ ± 0.93	11.93^cC^ ± 1.22	< 0.001
	**CHX**	22.74^bA^ ± 0.29	20.27^bB^ ± 1.17	16.64^bC^ ± 0.48	14.79^bD^ ± 1.02	< 0.001
***p*** **-value**		< 0.001	< 0.001	< 0.001	< 0.001	

Different lowercase letter indicates significant different within column, while different uppercase letter indicate significance within rows (adjusted p-value with Tukey HSD).

## Discussion

Effective and durable bond to different tooth substrates presents the optimum objective in adhesive dentistry [2]. Therefore, it is important to introduce dental adhesives that are capable of efficiently bonding to dentin with proper sealing of the prepared cavity margins to offer a tangible secondary caries resistance. Presently, SE bonding techniques that use mild-acidity monomers which create water-insoluble salts with dentin, such as the 10-MDP monomer, are considered as highly consistent dentin bonding agents [1, 2, 5]. On the other hand, the μSBS examination is valuable in determining any disparities in the bonded restorative system at various circumstances. Multiple interpretations could be obtained from small specimens from one tooth yielding more cordial stresses that could lead to minor data dispersal [25].

Irrespective to the applied decontamination protocols or the type of the adhesive systems employed, bonding to CAD showed inferior μSBS compared to SoD. This can be owed to the structural and morphological variations that occurred in the CAD substrate leading to poor hybridization and inferior bonding performance of the subsequently applied restorative system [26, 27]. Furthermore, the findings of the present study showed that μSBS was adversely influenced by saliva-blood contamination of SoD and CAD. Therefore, the first null hypothesis was rejected since all contaminated groups revealed less μSBS values compared to the control groups. Saliva contamination jeopardized the bonding performance, which could be owed to the salivary glycoproteins that might hinder the adhesive monomers diffusion into dentin collagen network [27]. Moreover, Lund et al. 2021 [28] reported a premature failure of the restorative system upon blood contamination that occurs during the bonding process following different decontamination approaches.

The second null hypothesis was rejected as the present study results showed that the saliva-blood contamination had significantly decreased μSBS even with employing ‘No-wait’ UBQ and the moisture‑tolerant universal adhesive ABU with both dentin substrates. This could be owed to the monomer competition throughout the dentin hybridization process. Moreover, the compositional monomer of both adhesives (Bis-GMA) might have degraded by the action of the hydrolytic enzymes of saliva-blood mixture with consequent inhibition of the adhesion process [27, 29]. These findings agreed with Oonsombat et al. 2003 [30] who reported a considerable μSBS decline from saliva and blood contamination for the applied universal adhesive. That could be related to the dentin bonding technique which relies primarily on hybridization and resin diffusion into the exposed collagen fibrils network. An efficient bonding of SE adhesives and universal adhesives relies upon the chemical reaction that occurs between dentin hydroxyapatite crystals and the adhesives functional monomer. Additionally, such considerable adhesion performance could be owed to elevated water and solvents content of some universal adhesives, which grants proper ionization of the incorporated acidic functional monomers, thus resulting in diffusion of the resin monomer into the full depth of the conditioned dentin [6, 31].

Nevertheless, the findings of the present study revealed that UBQ had an overall better bonding performance than ABU, which could be related to differences in the chemical composition of both evaluated universal adhesives [2]. While both tested adhesives were founded on 10-MDP monomer that provide chemical adhesion with dentin, the different catalysts and co-monomers could have dictated some discrepancies regarding the adhesive reactivity, adhesive film properties, and dentin bonding performance. Nevertheless, UBQ is a mild universal adhesive that has a pH of 2.3 while ABU is an ultra-mild universal adhesive that has a pH of 3.1, which might explain the better bonding performance of UBQ with lower pH value that might have enhanced its dentin bonding capacity compared to ABU. Thus, this might imply that UBQ was more capable in impregnating the smear layer and demineralizing the underlying dentin substrate. However, this finding was contradicted by Papadogiannis et al. 2019 [5] who reported non-significant SBS values for UBQ and ABU. Such contradiction could be owed to the storage time that was 24 hours in the present study, while Papadogiannis and co-workers 2019 [5] kept their specimens for one week before bond strength testing. Additionally, this contradiction could be also related to the test parameters discrepancies as they assessed the SBS with the notch-edge blade technique, while μSBS with the orthodontic wire loop method was employed for the current study.

Our finding revealed that adhesive contamination with saliva-blood mixture had negatively affected μSBS irrespective to the contamination stage of both universal adhesives (before and after light curing of the universal adhesives), thus the third null hypothesis was rejected as well. Contamination of the universal adhesives before light curing might alter the monomer conversion of the hydrophilic HEMA molecules which preserve the water inside the adhesive layer, thus restricting the chain growth occurs through adhesive polymerization, and yielding a plasticizing influence for the polymer and C = C oxidation [23]. Moreover, a compromised adhesive polymerization might occur due to the release of the by‑products and as a result of the elevated blood viscosity, which might minimize the penetration of the curing light. These findings were in accordance with Nair and Ilie, 2020 [29] who concluded that saliva contamination of the universal adhesives could be detrimental to the final bond strength. They reported that saliva contamination performed as a buffering layer that diminishes the etching ability of monomers of the universal adhesives, and leading to decreased monomer diffusion into the previously patent dentinal tubules and subsequently a decreased bond quality is clearly evident.

On the other hand, Yazici et al. 2007 [32] contradicted these findings as they conveyed an insignificant moisture outcome on the overall bond strength. This contradiction could be related to the variation in the chemical composition of the tested adhesives and the distinct testing parameters of both studies. Furthermore, salivary glycoproteins absorption onto the surface of the partially polymerized adhesive could prevent the co-polymerization that occurs on the top of the adhesive film [16]. The results of the current study showed that water rinsing, and adhesive rebond decontamination procedures were not able to restore the depleted μSBS at the different contamination stages of the universal adhesives to both dentin substrates, which could be owed to the inability of water rinsing to eliminate the saliva-blood contamination residues. This finding agreed with de Carvalho et al. 2010 [33] and Chang et al. 2010 [34] who demonstrated a failure in blood elimination with simple water rinsing, which could be related to the increased amounts of blood proteins macro molecules that resist water rinsing, and thus hinder the adhesive diffusion into the conditioned dentin surface.

EDTA and CHX cavity decontaminants were used in the present study among the tested decontamination procedures and accordingly the depleted bond strength was restored to some extent. The present results revealed higher μSBS values recorded for the decontamination procedure using CHX application for both universal adhesives contaminated before light curing for both dentin substrates implying a significant improvement in μSBS recovery. The contamination stage (before light curing of the adhesives) might have a significant effect in the bond strength enhancement due to absence of the air-inhibited layer at the top surface of the uncured adhesives, and therefore the glycoproteins will not adhere to the air-inhibited layer and develop a physical barrier that prevents proper bonding between the adhesive layer and the resin composite restoration. Moreover, the results of the present study showed that CHX was more efficient than EDTA to restore the depleted bond strength for the contaminated universal adhesives before and after light curing for both dentin substrates. In this context, Pashley et al. 2004 [35] concluded that CHX was able to prevent collagen fibers degradation at the resin/dentin interface via MMPs inhibition. This finding could be also related to CHX ability to remove the smear layer and decontaminate the dental cavity more effectively than EDTA [19]. Furthermore, such high μSBS value could be owed to CHX potential to eradicate loose organic remnants of the dental cavity walls [16, 36] thus enhancing the wettability of subsequent adhesive rebonding to the contaminated cavity walls [37]. Hence, water rinsing followed by CHX application might have been able to eliminate most contaminated uncured adhesive, giving better bonding chances with the dentin surface. This outcome could be supported by the findings of Meiers and Kresin [38] who concluded that CHX application altered the smear layer features by eliminating the loosely bounded smear layer remnants, which might enhance the infiltration of the acidic monomers of the adhesives applied in SE bonding mode. Likewise, the strong positive ionic charge of CHX might have combined with the phosphate groups of dentin surfaces, thus enhancing the dentin surface energy, and improving the adhesives-wettability of the dentin surfaces [37]. In addition, CHX might have increased the chemical bonding potential of both universal adhesives evaluated in the study owing to their 10-MDP acidic functional monomer content. Also, this could be related to the CHX probable stabilizing effect on the smear layer, which could turn it from a loosely attached, semi-permeable film into a tightly bonded layer [38]. Hence, declining the fluxes of the inherent dentinal fluids from the underlying moist dentin, leading to an increase in the bonding potential of the adhesives applied in SE bonding mode.

On the other hand, contamination of both adhesives after light curing might allow salivary glycoproteins and blood proteins to adhere to the air‑inhibited layer at the top of the adhesive surfaces creating a physical barrier that could hinder co‑polymerization between the resin composite and the adhesive [39]. Therefore, treating the contaminated adhesives after light curing with water rinsing followed by CHX application might be able to remove contaminant deposits and the uncured adhesive coat to some extent, allowing the successive adhesive rebond to produce more patent adhesive layer for enhanced dentin bonding, depending on the hydrophilicity of the compositional water-based primer of the universal adhesives which might permit the diffusion of such adhesives across the residual saliva and blood layer if persisted [40].

EDTA is a chelating organic compound which chelates calcium ions and removes the hydroxyapatite crystals selectively without deep penetration of the dentinal tubules. Moreover, EDTA is an inhibitor for the MMPs which was found to enhance the adhesive interface durability. It can dissolve dentin mineral content without causing collagen denaturation or affecting the organic matrix stability. Furthermore, it was found that EDTA could maintain or even improve dentin bond strength with different adhesive systems [19]. It was reported that upon using EDTA as a decontaminant agent after dentin contamination by a hemostatic agent, EDTA was capable of restoring the depleted bond strength of the examined SE adhesive to the adhesion level of the un-contaminated dentin [15]. Yet, additional research is required to assess the role of EDTA as a cavity decontaminant at different adhesive procedure contamination stages, which could be a promising substitute for cavity decontamination. The results of the current study demonstrated adhesive failures among the decontaminated CAD substrate for both universal adhesives (contaminated before and after light curing), though the mixed failure mode was major for the SoD and CAD decontaminated substrates in both universal adhesives (contaminated before and after light curing). In this context, the assessment of failure mode partly indicated an inadequate dentin bond strength at the composite-adhesive-dentin interfaces, which was contemplated by the prevalence of the adhesive fractures. This might be related to the presence of frequent areas of collagen fibrils that remained on the dentin surface and covered the dentin tubules [15], thus impeding the penetration of the universal adhesives into the demineralized dentinal tubules. However, the bond strength of SE adhesives based on 10-MDP and their mode of fracture at the dentin-adhesive interface might not be influenced by its demineralizing potential [41]. Nevertheless, the created three-dimensional network of the self-assembled nano layers on the demineralized dentin surfaces might cause mechanical strengthening at the hybrid layer-adhesive interface, making it more invulnerable to biodegradation [1, 42]. This was partly established in our results, as the groups with higher mean μSBS reported an increased percentage of mixed and cohesive failures besides the adhesive fracture pattern, whereas groups with inferior mean μSBS exhibited mostly adhesive failure irrespective to the adhesive type, contamination stages, and the applied decontamination protocols. This finding agreed with Nair and Ilie, 2020 [29] who concluded that the prevalent mode of fracture was the adhesive failure mode, regardless the aging and salivary contamination.

It is worth mentioning that one of the constraints of the current study was utilizing the μSBS testing procedure for bond strength evaluation, while the assessment of the viscoelastic properties of the restorative systems was not taken into consideration. Moreover, employing the tested universal adhesives only in SE mode. For more consistent results, the tested adhesives should have been used in ER mode as well. Dental cavity isolation is an ultimate aspect to be considered upon the application of the different bonding procedures. However, the cavity isolation protocol could be breached in several clinical situations, specifically when the operative site is near the gingival margin, with uncooperative patients or in case of malposed teeth. Under these circumstances, the cavity contaminants would hinder the adhesive diffusion into the dentinal tubules, thus reducing the bonding quality to the teeth substartes [29]. Hence, decontamination of the dental cavities became a valuable step prior to the regular restorative procedures, which could be performed through cleaning the dental cavities using different antimicrobial agents before adhesive systems application, to ensure proper elimination of the different cariogenic bacteria as well as different contaminants. Thus, avoiding secondary caries formation and/or failure of the adhesive restoration due to the compromised bond strength to the different dentin substrates.

A variety of products is accessible for cavity decontamination before dentin bonding, yet only a little have been assessed to a suitable level proving clinical and in vitro sustainability. One can indicate future validation of the present study findings utilizing alternate assessment techniques such as fracture resistance or toughness. Moreover, further clinical investigations should be performed to assess the viability and role of CHX and EDTA as well as other cavity decontaminants of the different dentin substrates, as it was earlier proven that bonding to CAD substrate is complex and more clinically relevant than bonding to SoD. Last but not least, further studies are recommended to evaluate the long-term efficacy of various decontamination protocols on the bond strength to the different dentin substrates at different storage media and conditions to simulate the clinical situations as closely as possible.

## Conclusions

Within the limitations of this in vitro study, it can be concluded that bonding to CAD is more complex and inferior to bonding to SoD. Saliva and blood contamination can dramatically affect the bond quality of different dentin substrates. Therefore, field isolation is mandatory. CHX is a potent cavity decontaminant that is able to restore and maintain the bond strength of the different dentin substrates, meanwhile EDTA presents a promising substitute. Thus, regular use of cavity decontaminants is highly advised in daily practice to avoid possible detrimental effect of accidental cavity contamination. ‘No-wait’ UBQ adhesive showed better bonding performance than ABU to both dentin substrates even after blood-saliva contamination. The contamination stage during the bonding procedure of the tested universal adhesives had a considerable influence on bonding performance to the sound and caries-affected dentin.

## Data Availability

The datasets generated during and/or analyzed during the current study are not publicly available due to institutional policy but are available from the corresponding author on reasonable request.

## Abbreviations

μSBS: Micro shear bond strength
ABU: All-Bond-Universal
ANOVA: Analysis of variance
Bis-GMA: Bisphenol A diglycidyl methacrylate
CAD: Caries-affected dentin
CHX: Chlorhexidine
EDTA: Ethylenediaminetetraacetic acid
ER: Etch-and-Rinse
HEMA: Hydroxyethyl methacrylate
MDP: Methacryloyloxydecyl dihydrogen phosphate
MMPs: Matrix metalloproteinases
MPa: Megapascals
MREC: Medical Research Ethical Committee
N: Newtons
NRC: National Research Centre
SD: Standard deviation
SE: Self-etch
SiC: Silicon carbide
SoD: Sound dentin
TEGDMA: Triethylene glycol dimethacrylate
UBQ: Clearfil Bond Universal Quick
UDMA: Urethane dimethacrylate
